# The story of Biology Open: a conversation with past and present Editors-in-Chief

**DOI:** 10.1242/bio.061897

**Published:** 2025-02-26

**Authors:** Saanjbati Adhikari, Alejandra Clark, Rachel Hackett

**Affiliations:** ^1^ Features Editor at The Company of Biologists; ^2^ Managing Editor of Biology Open; ^3^ Managing Editor of Disease Models & Mechanisms

**Figure:**
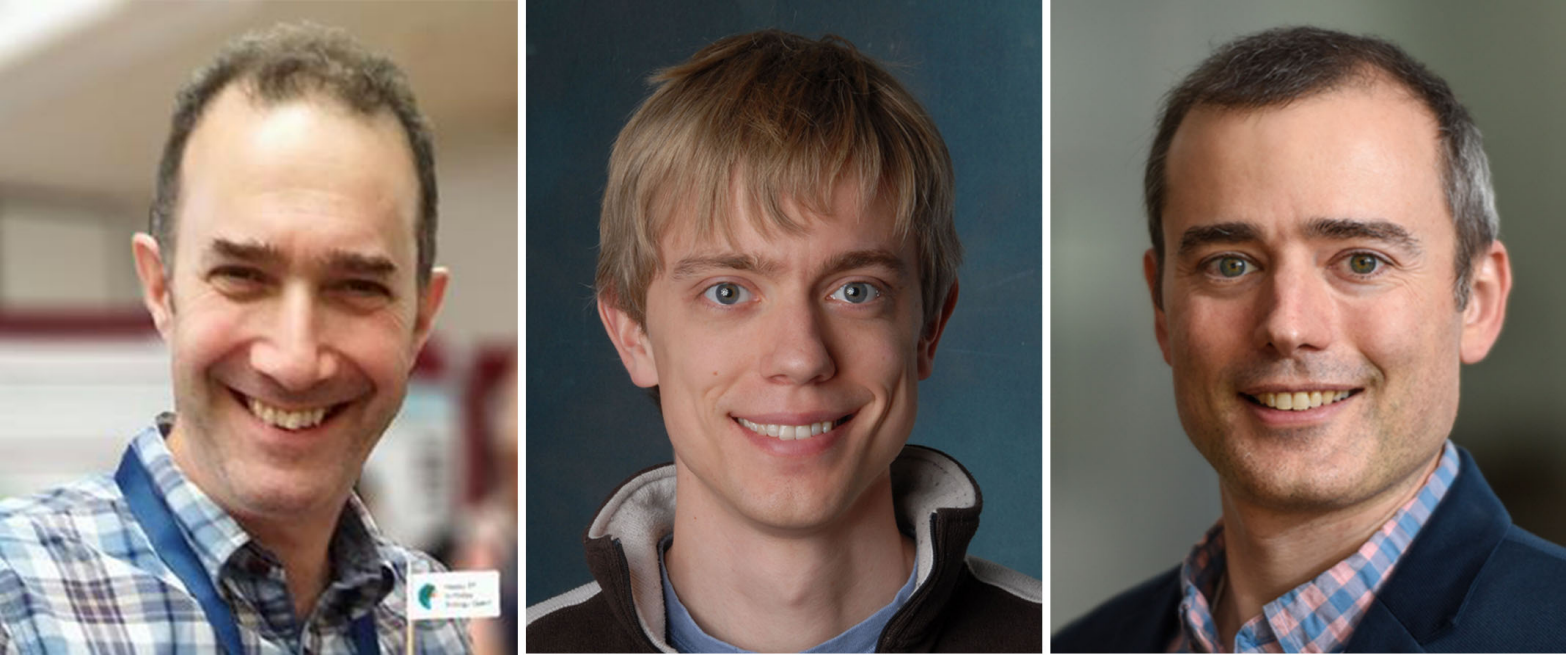
Biology Open Editors-in-Chief (left to right): Jordan Raff (2012–2018), Steven Kelly (2018–2023) and Daniel Gorelick (2023–present).

The Company of Biologists is celebrating its 100th birthday in 2025. As a not-for-profit publisher run by distinguished practising scientists, we publish five specialist high-quality journals: Development, Journal of Cell Science, Journal of Experimental Biology, Disease Models & Mechanisms and Biology Open. This Editorial looks back to the founding of Biology Open and its evolution under the guidance of three successive Editors-in-Chief, all leading researchers and widely respected within the biological sciences community.

In 2010, the Board of Directors of The Company of Biologists approved a proposal to launch an Open Access (OA) online-only journal that would offer a rapid-publication service to authors and reduce the pain to publish. The subsequent year, it was decided that the journal would be called Biology Open (BiO), with Jordan Raff (University of Oxford, UK) as its inaugural Editor-in-Chief (EiC), along with John Speakman (University of Aberdeen, UK) as deputy EiC and a team of nine Editors (to handle submissions to the journal), who were all leading researchers from around the world.

The first issue of BiO was published in January 2012 with the primary objective of publishing “good-quality, sound research, without attempting to judge impact or novelty”. In his introductory Editorial, Raff wrote, “It will be up to the scientific community to decide, after publication, on the importance of each paper” (Raff, 2012).

When the journal celebrated its 10th birthday, then-EiC Steven Kelly (University of Oxford) wrote an Editorial featuring a timeline of the journal since its launch. “I believe BiO will still be pioneering initiatives to support the biological sciences community and will still be focused on finding ways to support the career development of early-career researchers”, shared Kelly (Kelly, 2022).

Here, Jordan Raff, founding EiC from 2012 to 2018, Steve Kelly, EiC from 2018 to 2023 and Daniel Gorelick (Baylor College of Medicine, USA), current EiC, share their experiences of being involved with the journal and their aspirations for the future of BiO.

## Why did you take on the role of EiC, and what were/are your hopes and aspirations for the journal?

**JR:** I had been a Director at The Company of Biologists since 2003, and we were constantly discussing ways in which the Company can adapt to the rapidly changing publishing environment. Several of us became enthused with the idea of creating a new journal that could capture the Research Articles that include sound findings and experiments but are rejected by the Company's flagship journals (Development, Journal of Cell Science, Journal of Experimental Biology and Disease Models & Mechanisms), either due to being beyond the scope of the journals or due to the lack of ‘advanced’ findings. I spearheaded the launch of BiO; so, when the Company's Board of Directors asked whether I would be interested in being the first EiC of the journal, I couldn't refuse. I really hoped it would be a win–win situation for both the Company and the scientists who submit articles to the Company's journals.

**SK:** Science helps us understand the world and our place in it. We communicate advances in our understanding of science through teaching, conferences, social media, and a multitude of other platforms that continuously and rapidly evolve. However, the bedrock of science, and thus the platform on which all of this communication is built, are scientific papers. I felt that this foundation had not really changed in a hundred years, certainly not at the pace in which science communication was changing, and I felt that I wanted to try do something to improve this. I was already an Editor at BiO and when the opportunity to step up and become EiC came along, I was excited to take on the challenge and see if I could help guide the evolution of scientific publishing.

**DG:** I became EiC because I am passionate about improving scientific publishing, particularly the peer review process. I saw the role as an opportunity to implement ideas that could make peer review faster, fairer and more transparent. I have also loved working with the incredible team at The Company of Biologists, especially BiO's in-house team: Alejandra Clark, Ania Crowther, Laura Tolhurst and Sue Chamberlain. Connecting with the EiCs of BiO's sister journals and the Company's Board of Directors – accomplished scientists I probably wouldn't have had the privilege to interact with otherwise – has been inspiring and exciting. My goal is to keep pushing the boundaries to improve the quality and transparency of peer review while maintaining the high publishing standards of the journal. I want BiO to be known for cost-effective, high-quality peer-review with rapid turnaround – publishing manuscripts where the conclusions are supported by the data, irrespective of impact.

## What were/have been the main achievements and the biggest challenges of the journal during your tenure?

**JR:** I definitely think our main achievement was that we survived! We had not realised just how many other publishers would be launching journals with a similar ethos. I guess this proves it wasn't a completely stupid idea, but it meant the competition was fierce. The biggest challenge was that we got many more direct submissions (i.e. papers that had not previously been submitted to one of our flagship journals) than we anticipated. This meant our team of Editors often had much more work to do than we had suggested to them when we asked them to join. Luckily, they were a tremendous team and they really rose to the challenge.

**SK:** I would say that there were two main impacts: the first is the suite of initiatives we launched to support early-career researchers (ECRs), from internships to our ‘Future Leader’ and ‘A Year at the Forefront’ writing programmes, to our funding of ECR-driven conferences and events. These created a platform and career-enhancing opportunities for ECRs, and also allowed them to experiment with publication and scientific meetings. These were hugely insightful for me, and I think the whole team learned and developed in response to the proposals and articles we supported. The second thing I think has made an impact was the recent biopositive initiative taken by The Company of Biologists, called The Forest of Biologists (Moulton and Freeman, 2023). I am passionate about the natural world and I wanted to find a way for scientific publishing to make a positive impact not only on our understanding of the natural world, but also do something to enhance and sustain its endless forms most beautiful and most wonderful. The idea was super simple: what if we could plant a tree for every paper we published and restore degraded habitats for every review we received? I had initially planned to do this just for BiO, but the whole Company got on board and we ended up rolling it out company-wide. I am hugely grateful to our collaborators at the Woodland Trust who helped make this a reality and also enabled us to use the project to further advance science communication and education concerning forests and biodiversity. It has been incredibly rewarding to watch the forest grow and see the impact it has had on people, the landscape and biodiversity.

There were lots of challenges during my time. We saw the emergence and meteoric rise of papermills, we witnessed the reproducibility crisis, and we struggled to be noticed in the shadow of the “cash and carry” mega publishers. However, I think the biggest challenge was figuring out how to make a positive impact on science publishing. How do we take the 100+ year-old academic publishing system and find a way to make it work better for our authors, reviewers and readers. I think this was the hardest thing to do and I hope we moved the needle a little bit.

**DG:** Two initiatives stand out: first, we introduced a rubric to make our peer review and decision-making process more transparent, which provides clear expectations for reviewers and authors, and ensures consistent decision-making. Second, we recently launched an exciting and innovative peer review experiment, which aims to provide authors with a decision and reviews within seven business days of submission and also compensates reviewers for their valuable time and expertise. This is a bold attempt to address the low quality and delays often associated with peer review, and it's been an exciting innovation for BiO. However, implementing this experimental system also comes with its own set of unique challenges. From drafting legal contracts that outline reviewer expectations to creating workflows with extremely tight turnaround times, there were many hurdles. Thankfully, our Editors and peer reviewers have been incredibly supportive and have risen to the challenge. A special shout-out goes to Alejandra Clark, whose hard work and dedication were instrumental in making it happen.

## What was/has been the most memorable moment whilst EiC at BiO?

**JR:** My most memorable moment was the day BiO was launched. It was at a society conference (British Society for Cell Biology and British Society for Developmental Biology). I walked into the exhibition hall and saw a big table with several posters and promotional material trumpeting the launch of a new journal, which was called Open Biology. My first instinct was that we had made a terrible mistake and printed all this paraphernalia with the name the wrong way around, so I was mortified. I was even more mortified, however, when I realised that the table was not being run by The Company of Biologists, but by the Royal Society, who were also launching a new journal, called Open Biology, the very same day! Looking back, it seems very funny, but I can promise you I wasn't laughing much on the day. Luckily, both journals have gone on to be successful in their own ways and, if I am honest, I still sometimes get their names the wrong way around.

**SK:** I think my most memorable moment was my first Board meeting as EiC. I sat nervously in front of a room full of scientific giants and told them about my plans for the journal. I was expecting some resistance, or at least a fair amount of scepticism, but what I got was encouragement and support. If I had my time again, I would probably try to make more radical changes and see if I could raise a few eyebrows among the Board.

**DG:** One standout moment was the journal Editors' meeting in 2024. It was fantastic to discuss challenges and witness the enthusiastic support for our new experimental peer review process. Then, in October 2024, we reviewed the results of the initial three-month trial of the experiment and realised it was working. Every manuscript in the experiment was reviewed on time, and the decisions met our high standards. Seeing this ambitious idea succeed was incredibly satisfying, although we have more to do to make sure that this experimental process is a success for every manuscript submitted to BiO.

## Jordan and Steve, how did you perceive BiO's state and direction when you left the role?

**JR:** Although it was hard work, I really loved my time at BiO. Rachel Hackett, former Managing Editor of BiO, was fantastic, and all my fellow Editors and the in-house team at the Company were great fun to work with. But after seven years at the helm, I realised that I was running out of energy. I told Steve Kelly, an Editor at the time, that I was thinking of stepping down and I could see he was still brimming with enthusiasm and ideas. I immediately knew that he would be a potential successor who would take things forward in a way I just couldn't do anymore; and the rest is history. Although I don't know Daniel Gorelick personally, I know that the Company's Board of Directors (who oversee the appointment of new EiCs, usually following a stringent recruitment process) and Steve felt the same way about him. So, I remain very optimistic for BiO and The Company of Biologists, even though the scientific publishing ecosystem remains extremely volatile and challenging.

**SK:** After adding a few miles and making a few modifications to BiO, I hope I left it in as good a state as when I received it from Jordan. Dan has taken the journal in a new and exciting direction that builds on its legacy of assisting with the evolution of scientific publishing.

## Daniel, how do you perceive BiO's future in the coming 5–10 years? What would you specifically like to achieve as an EiC?

**DG:** I would love to see our new peer review model take off – not just at BiO, but also at the other Company journals and beyond – to scientific publishing as a whole. Imagine if the standard for peer review in every field was paying reviewers and receiving high-quality reviews within a week! I also want to make peer review even more transparent. Ideally, all manuscripts would originate as preprints before submission, and peer review reports would be published alongside every manuscript. For manuscripts that BiO accepts, the reviews would be published along with the article. For those we reject, the reviews could be posted on the preprint server alongside the manuscript. This would make the peer review process more relevant and valuable to the scientific community.

## Concluding remarks

As the Company's newest journal, BiO aims to be a home for all biologists. Our ethos is to benefit the research community by rapidly disseminating rigorous and reliable science in a transparent manner. Backed by our Directors and a dedicated journal team, we are committed to innovation and experimentation as we look forward to shaping the future of scientific publishing.

The Company of Biologists is marking its 100-year anniversary this year. Our landmark celebrations include the Biologists @ 100 conference that we are hosting at ACC Liverpool, UK from 24–27 March 2025. This unique conference will combine the Spring Meetings of the British Society for Cell Biology and the British Society for Developmental Biology, a Journal of Experimental Biology Symposium, a one-day Disease Models & Mechanisms meeting and a Society for Experimental Biology satellite symposium. We hope to see you there to celebrate this extraordinary Company.

Our EiC, Daniel Gorelick, Editors and the expert members of the Editorial Advisory Board are invaluable in helping BiO publish timely and high-quality Research Articles and Reviews. Additionally, our Cambridge-based dedicated in-house team, led by our Managing Editor, Alejandra Clark, supports our Editors, readers, reviewers and authors (Box 1). We thank them all for their hard work, dedication and expertise.

**Box 1. BiO's current in-house team**

The Cambridge-based dedicated in-house team is overseen by our Managing Editor, Alejandra Clark, who works closely with BiO's EiC, Daniel Gorelick, to implement key strategic decisions. Our Journal Editorial Coordinators, Ania Crowther and Laura Tolhurst, meticulously copycheck each issue, correct proofs and ensure proper indexing and archiving. Finally, our Senior Editorial Administrator, Sue Chamberlain, supports authors and reviewers throughout the crucial steps of manuscript submission and peer-review.

The Production team provides graphical and quality control checks in publishing articles and technical support in maintaining our systems, and The Company of Biologists' Ethics Coordinator handles cases of publishing ethics concerns. The team thanks our colleagues – past and present – for their contributions.
